# Prevalence and patterns of prenatal use of traditional medicine among women at selected harare clinics: a cross-sectional study

**DOI:** 10.1186/1472-6882-12-164

**Published:** 2012-09-27

**Authors:** Dudzai D Mureyi, Tsitsi G Monera, Charles C Maponga

**Affiliations:** 1 Drug and Toxicology Information Service, University of Zimbabwe School of Medicine, Parirenyatwa Hospital, Harare, Zimbabwe

**Keywords:** Traditional medicine, Prenatal, Prevalence

## Abstract

**Background:**

Prenatal use of traditional medicine or complementary and alternative medicine is widespread globally despite the lack of evidence of the effectiveness of these therapeutic options. Documentation on the prevalence and patterns of this maternal practice in the Zimbabwean setting was also lacking.

**Methods:**

A cross sectional survey of 248 women at selected health centres in Harare was carried out to address the need for such data using an interviewer-administered questionnaire.

**Results:**

Fifty-two (52%) (95% C.I. 44%-60%) of the participants reported to have used at least one traditional medicine intervention during the third trimester of their most recent pregnancy to induce labour, avoid perineal tearing and improve the safety of their delivery process. The study found prenatal use of traditional medicine to be significantly associated with nulliparity and nulligravidity. Such practice was also significant among participants residing in a particular high density suburb located in close proximity to informal traders of traditional medicines. Prenatal traditional medicine use was not significantly linked to experiencing an obstetrics-related adverse event. Instead, participants who reported not using any traditional medicine during pregnancy reported experiencing significantly more adverse events, mainly perineal tearing during delivery.

**Conclusions:**

The practice of prenatal use of traditional medicine was significant in the study setting, with a prevalence of 52%. A variety of products were used in various dosage forms for differing indications. Nulliparity, nulligavidity and possible accessibility of these products were the factors significantly associated with prenatal use of traditional medicine. Prenatal use of traditional medicine was not significantly associated with any obstetric adverse event.

## Background

Traditional medicine (TM) is defined as, “the sum total of knowledge, skills and practices based on the theories, beliefs and experiences indigenous to different cultures that are used to maintain health, as well as to prevent, diagnose, improve or treat physical and mental illnesses” [1]. Prenatal use of TM is widespread globally despite the lack of evidence of its safety. Some literature even suggested the lack of safety of prenatal use of TM [2], [3]. Investigators in a clinical trial testing the effectiveness of early application of a non-pneumatic anti-shock garment for obstetric haemorrhage (NASG) at a satellite health facility in Zimbabwe hypothesized that the prenatal use of some TMs especially *Cannabis sativa* was linked to post-partum haemorrhage and uterine rupture. The investigators requested the authors to find out the mechanism of action of these TMs. A preliminary cross-sectional study to validate these anecdotal claims was required before any in-vitro studies to discover the mechanism of action of these TMs could be designed. The objectives of this study were to; 1) determine the prevalence of prenatal use of TM within a sample of women drawn from selected clinics, 2) find out the most commonly used traditional medicines, 3) determine which factors were significantly associated with the practice, and 4) determine whether prenatal use of TM was significantly associated with obstetric-related adverse events such as perineal tearing. Identification of the most commonly used traditional medicines would then form the basis for further studies to address the query that had been raised by the NASG team.

## Methods

**Figure 1:**
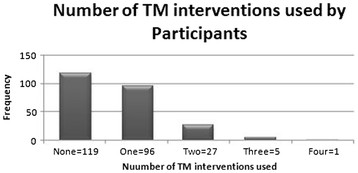
**Data Collection Tool** .

A cross-sectional study design was chosen as the best observational method to determine prevalence and associations [4]. Twelve Harare public maternity clinics from where participants of the NASG clinical trial were being drawn were selected as the study sites. Participants were drawn from women who presented at the study sites for the mandatory six weeks postnatal reviews between 1 November 2010 and 31 March 2011. An interviewer-administered questionnaire (see Figure 1), was used to collect data from eligible participants after the study’s working definition of “Traditional Medicine” was explained to them. This questionnaire was formulated by modifying the one used by Rahman and co-workers [5]. The questionnaire was then piloted among 20 participants before being adopted as the study’s data collection tool. The sample size of at least 246 was calculated to enable estimation of the proportion of women who used traditional medicines during the prenatal period at a 95% confidence level, allowing for a 5% margin of error. This was after the pilot study revealed that 80%of the population of interest utilised traditional medicines during the prenatal period. Eligible participants were Shona or English speaking women visiting the study sites for their six weeks postnatal reviews during the specified period, who were able to give their full informed consent. The six-week period was regarded as long enough to allow the participants to recover from the birthing experience yet short enough post-delivery to avoid recall bias among the participants. At least 20 participants were recruited from each site. Before sampling was done at any given site, the investigator met all the participants, carried out a self-introduction, and informed the participants about the interview’s concern with their experiences during their latest pregnancy but did not disclose the exact study title. This was done to minimise recruitment bias. After the meeting, the women were invited into a private room one at a time to receive more details about the study objectives and the written informed consent process. Those who gave their full informed consent went on to complete the interviewer-administered questionnaire while those who could not give their consent were allowed to leave the room. Collected data were entered on a Microsoft Excel 2007^©^ spreadsheet for mathematical operations such as calculation of totals, means and proportions and for construction of tables and charts. Stata Version 8.0™ was used for further statistical analysis. Chi Square tests were performed to test the significance of associations and a p-value of <0.05 was considered to be significant. This study was approved by the Harare City Council Department of Health and was deemed ethically sound by the Joint Parirenyatwa Hospital and University of Zimbabwe College of Health Sciences Research Committee (JREC), Reference Number; JREC/104/11.

## Results

### Demographic characteristics

Participants’ ages ranged from 16–50 years old and average age was 26 years (Standard deviation = 6.1 years). All participants were married and all had received at least primary level education. All except three participants cited Christianity as their religion. The rest of the characteristics of the study participants are summarised in Table 1 along with their respective p-values (as indicators of significant association to prenatal use of TM).

**Table 1:** **Demographic characteristics of thestudy participants**

**Characteristic**	**Frequency n (%) n** **= 248**			**P value**
	**Users = 129 (52.02)S.E (3.17%)**	**Non-users = 119 (47.98)SE (3.17%)**	**Total = 248 (100)**	
**Age (years)**				
**<20**	15 (6.05) S.E (1.51**%**)	7 (2.82) S.E (1.05**%**)	22 (8.87) S.E (0.57**%**)	P = 0.113
**20-25**	60 (24.2) SE (2.72**%**)	37 (14.91) SE (2.26**%**)	97 (39.11) SE (3.1**%**)	P = 0.021
**26-30**	30 (12.09) SE (2.07**%**)	41 (16.62) SE (2.36**%**)	71 (28.63) SE (2.87**%**)	P = 0.183
**31-40**	21 (8.47) SE (1.77**%**)	30 (12.09) SE (2.07**%**)	51 (20.56) SE (2.57**%**)	P = 0.206
**>40**	3 (1.21) SE (0.69**%**)	4 (1.61) SE (0.8**%**)	7 (2.82) SE (1.51**%**)	P = 0.714
**Level of Education**				
**None**	0 (0)	0(0)	0 (0)	
**Primary**	7 (2.82) SE (1.05**%**)	6 (2.42) SE (0.98**%**)	13 (5.24) SE (1.41**%**)	P = 0.7774
**Secondary**	114 (46) SE (3.16**%**)	99 (39.89) SE (3.11**%**)	213 (85.89) SE (2.21**%**)	P = 0.244
**Tertiary**	8 (3.22) SE (1.12**%**)	14 (5.65) SE (1.47**%**)	22 (8.87) SE (0.57**%**)	P = 0.205
**Employment**				
**Housewife**	83 (33.47) SE (3.00**%**)	63 (23.4) SE (2.69**%**)	146 (56.87) SE (3.14**%**)	P = 0.094
**Self-Employed**	22 (8.87) SE (0.57**%**)	32 (12.9) SE (2.13**%**)	54 (21.77) SE (2.62**%**)	P = 0.193
**Formally employed**	13 (5.24) SE (1.41**%**)	20 (8.07) SE (1.73**%**)	33 (13.31) SE (2.28**%**)	P = 0.216
**Student**	11 (4.44) SE (1.31**%**)	4 (1.61) SE (0.8**%**)	15 (6.05) SE (1.51**%**)	P = 0.107
**Parity**				
**1**	58 (23.39) SE (2.69**%**)	29 (11.69) SE (2.04**%**)	87 (35.08) SE (3.03**%**)	P = 0.004
**2**	45 (18.15) SE (2.45**%**)	45 (18.15) SE (2.45**%**)	90 (36.29) SE (3.05**%**)	P = 1.000
**3**	20 (8.06) SE (1.73**%**)	36 (14.52) SE (2.24**%**)	56 (22.58) SE (2.65**%**)	P = 0.044
**4**	5 (2.02) SE (0.89**%**)	7 (2.82) SE (1.05**%**)	12 (4.84) SE (1.36**%**)	P = 0.585
**5**	1 (0.41) SE (0.41**%**)	1 (0.41) SE (0.41**%**)	2 (0.81) SE (0.57**%**)	P = −*
**6**	0 (0)	1 (0.41) SE (0.41**%**)	1 (0.40) SE (0.41**%**)	P = −*
**Gravida**				
**1**	52 (21) SE (2.59**%**)	23 (9.27) SE (1.84)	75 (30.24) SE (2.92**%**)	P = 0.002
**2**	41 (16.5) SE (2.36**%**)	43 (17.34) SE (2.4**%**)	84 (33.87) SE (3.00**%**)	P = 0.855
**3**	27 (10.89) SE (1.98**%**)	40 (16.13) SE (2.34**%**)	67 (27.02) SE (2.82**%**)	P = 0.108
**4**	8 (3.23) SE (1.12**%**)	10 (4.03)SE (1.25**%**)	18 (7.26) SE (1.65**%**)	P = 0.555
**5**	1 (0.41) SE (0.41**%**)	1 (0.41) SE (0.41**%**)	2 (0.81) SE (0.57**%**)	P = −*
**6**	2 (0.81) SE (0.57**%**)	0 (0)	2 (0.81) SE (0.57**%**)	P = −*
**Tribe**				
**Zezuru**	60 (24.19) SE (2.72**%**)	55 (22.18) SE (2.64**%**)	115 (46.37) SE (3.17**%**)	P = 0.668
**Karanga**	18 (7.26) SE (1.65**%**)	14 (5.64) SE (1.47**%**)	32 (12.90) SE (2.13**%**)	P = 0.558
**Maungwe**	12 (4.84) SE (1.36**%**)	11 (4.43) SE (1.31**%**)	23 (9.27) SE (1.84**%**)	P = 0.661
**Manyika**	9 (3.63) SE (1.19**%**)	14 (5.64) SE (1.47**%**)	23 (9.27) SE (1.84)	P = 0.303
**Ndebele**	5 (2.02) SE (0.89**%**)	4 (1.61) SE (0.8**%**)	9 (3.63) SE (1.19**%**)	P = 0.720
**Korekore**	13 (5.24) SE (1.41**%**)	11 (4. 44) SE (1.31**%**)	24 (9.68) SE (1.88**%**)	P = 0.696
**Ndau**	2 (0.81) SE (0.57**%**)	3 (1.21) SE (0.69**%**)	5 (2.02) SE (0.89**%**)	P = 0.661
**Toko**	7 (2.82) S.E (1.05**%**)	4 (1.61) SE (0.8**%**)	11 (4.44) SE (1.31**%**)	P = 0.370
**Foreigner**	3 (1.21) SE (0.69**%**)	3 (1.21) SE (0.69**%**)	6 (2.42) SE (0.98**%**)	P = 1.000
**Area of Residence**				
**Budiriro**	11 (4.44) SE (1.31**%**)	9 (3.63) SE (1.19**%**)	20 (8.06) SE (1.73**%**)	P = 0.656
**Mbare**	15 (6.05) SE (1.51**%**)	5 (2.02) SE (0.89**%**)	20 (8.06) SE (1.73**%**)	P = 0.046
**Glen View**	12 (4.84) SE (1.36**%**)	8 (3.22) SE (1.12**%**)	20 (8.06) SE (1.73**%**)	P = 0.380
**Hatcliffe**	11 (4.44) SE (1.31**%**)	9 (3.63) SE (1.19**%**)	20 (8.06) SE (1.73**%**)	P = 0.656
**Highfield**	24 (9.68) SE (1.88**%**)	19 (7.66) SE (1.69**%**)	43 (17.34) SE (2.4**%**)	P = 0.443
**Kambuzuma**	12 (4.84) SE (1.36**%**)	8 (3.22) SE (1.12**%**)	20 (8.06) SE (1.73**%**)	P = 0.380
**Kuwadzana**	9 (3.63) SE (1.19**%**)	11 (4.44) SE (1.31**%**)	20 (8.06) SE (1.73**%**)	P = 0.656
**Mabvuku/Tafara**	6 (2.42) SE (0.98**%**)	14 (5.64) SE (1.47**%**)	20 (8.06) SE (1.73**%**)	P = 0.097
**Mufakose**	10 (4.03) SE (1.25**%**)	15 (6.05) SE (1.51**%**)	25 (10.08) SE (1.73**%**)	P = 0.327
**Dzivarasekwa**	6 (2.42) SE (0.98**%**)	14 (5.64) SE (1.47**%**)	20 (8.06) SE (1.73**%**)	P = 0.097
**Warren Park**	13 (5.24) SE (1.41**%**)	7 (2.82) S.E (1.05**%**)	20 (8.06) SE (1.73**%**)	P = 0.199

* Not enough observations to test for significance. Table 1 shows the demographic characteristics of the study population; their age, level of education and employment status according to whether they were prenatal users of TM interventions (users) or non-users.

SE = Standard Error.

### Prevalence

In total, 248 participants at the twelve study sites were interviewed, and of these, 129, reported that they employed at least one traditional medicine-based intervention during pregnancy. The prevalence of prenatal use of TM within the study population was 52% (n = 129, 95% C.I. = 44%- 60% and Standard error of 3.17%).

### Patterns of use

**Figure 2:**
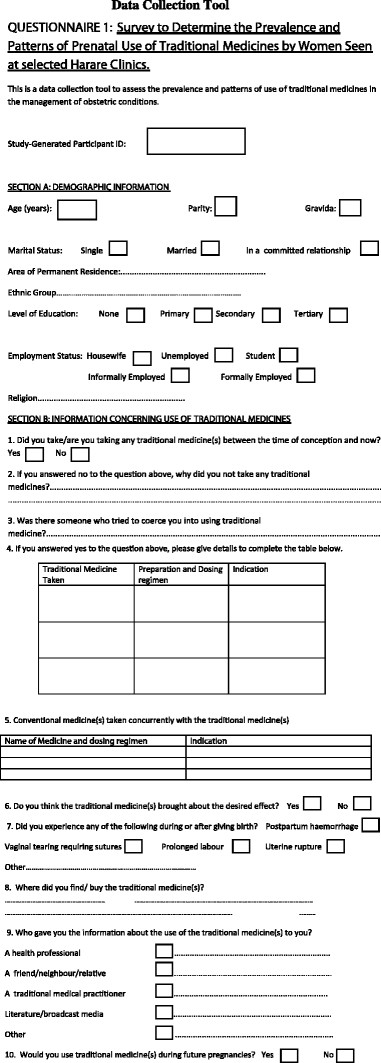
**The number of womenwho used different numbersof TMs concurrently duringtheir pregnancies.** Figure 2 shows the number of women who used different numbers of traditional medicine interventions during their pregnancy. Among users, the majority (n = 96) used only a single intervention, 27 users used two interventions concomitantly while 5 employed three interventions. Only one user reported the use of four interventions during a single pregnancy.

The most commonly used traditional medicine interventions, their dosing regimens and the indications for use are summarised in Table 2. Users reported use of at least one intervention but several used more than one as shown in Figure 2. Among the users, the majority (n = 96) used only a single intervention, 27 users used two interventions concomitantly while 5 employed three interventions. Only one user reported the use of four interventions during a single pregnancy. Characteristics of pregnant women which were significantly associated with prenatal use of TM were; being in the 20–25 age group (p = 0.021), nulliparity (p = 0.004), nulligravidity (p = 0.002), and residing in the Mbare high density neighbourhood (p = 0.046). Tribe of origin, religion and level of education and employment status were not statistically significantly associated with prenatal use of TM. This study revealed that women regarded perineal tearing during parturition as an adverse and undesirable event. Widening of the birth canal to prevent perineal tearing was therefore the most common indication for using traditional medicine during pregnancy. Perineal tearing was reported to have been experienced by 140 participants. Other perinatal adverse events reported were; postpartum haemorrhage (n = 2), retained placenta (n = 1), breech birth (n = 1), prolonged labour (n = 2), eclampsia (n = 1) and erratic postnatal bleeding (n = 1). Interestingly, it was noted that the non-use of traditional medicine was significantly associated with experiencing at least one adverse event, (p = 0.001). Use of TM was not found to be significantly associated with adverse events (p = 0.339). Other conditions which predisposed women to adverse events (mainly perineal lacerations), were nulliparity (p = 0.012) and nulligravidity (p = 0.029).

**Table 2:** **Patterns of prenatal useof TM within thestudy population**

**Intervention**	**Dosing regimen**	**Indication(s)**
**Holy water**	Drunk in 3^rd^ trimester or throughout pregnancy in amounts which are at the patient’s discretion.	· For protection against evil spirits
		· For a safe and uneventful delivery
**Soil from burrowing mole**	Soil is mixed with water and the supernatant is drunk in varying amounts in the 3^rd^ trimester	For widening of birth canal to avoid perineal tearing
**Pouzolzia mixta *(nhanzva)***	Aqueous extract of root is applied intravaginally in 3^rd^ trimester, usually with manual exercises to dilate the birth canal.	For widening of birth canal to avoid perineal tearing
**Elephant Dung**	Dung is mixed with water and variable amounts of the supernatant are drunk during the 3^rd^ trimester	For widening of birth canal to avoid perineal tearing
**Unknown herbs/concoctions**	Unspecified amounts are taken during the 3^rd^ trimester or even during labour	· For widening of birth canal
		· For labour induction
**Manual exercises**	Performed by hand lubricated with oil, soap, warm water or Poulozozia mixta, in 3^rd^ trimester	Dilation of birth canal to avoid tearing
**Abelmoschus esculuntus *(Okra/derere)***	Cooked and taken orally in the 3^rd^ trimester.	· Nutrition
		· Widening of birth canal
**Hot water/steam baths**	Taken inconsistently during the 3^rd^ trimester	Birth canal dilatation
**Cannabis sativum *(mbanje)***	Aqueous extract is drunk as soon as labour commences	Labour induction to speed up the labour
**Dicerocaryum zanguebarium *(ruredzo)***	Administered both orally and intravaginally in the 3^rd^ trimester	Widening of birth canal to avoid tearing
**Castor oil**	5 ml taken at night daily in 3^rd^ trimester	For constipation and labour induction
**Rooibos Tea**	Taken frequently throughout the pregnancy	For widening of birth canal to avoid tearing
Other*	Varying methods	Widening of birth canal and labour induction

*Other interventions used by participants: “church-made” coffee (n  = 1), Albizia amara *(muora)* (n  = 1), Terminalia sericea *(mususu)* (n  = 1), hare droppings (n  = 1), warm water drinks (n  = 4), ricinus communis root (n  = 1), avocado seeds (n - 1), apple seeds (n  = 1), Table 2 shows the traditional medicine interventions used and the dosing regimens employed, the stage of pregnancy in which the intervention was used and the number of participants (n) who reported using the intervention. NB: * denotes “Other interventions” used by participants: “church-made” coffee (n = 1), Albizia amara *(muora)* (n  = 1), Terminalia sericea *(mususu)* (n  = 1), hare droppings (n  = 1), warm water drinks (n = 4), ricinus communis root (n = 1), avocado seeds (n-1), apple seeds (n  = 1).

Almost all TM interventions were employed beginning at the onset of the 3^rd^ trimester and doses and dosing intervals were rarely standardised. The most commonly used TMs (reported to have been used by N > 10) were; holy water, soil burrowed by moles, Pouzolzia mixta root (*nhanzva*), elephant dung, cocktails of unknowns herbs and manual exercises with various lubricants.

## Discussion

This study detected 52% prevalence of prenatal use of TM with a standard error of 3.17%, lower than the study’s acceptable margin error of 5%. This prevalence value is within the same range as the one reported by Rahman and co-workers from similar work in Tumpat district, Malaysia in 2008 (50.4%), [5] by Kalder et al., in Germany in 2010 (50.8%) [6], by Cuzzolini and colleagues in Italy in the year 2010 (46.7%) [7] and by Mabina et al. in South Africa in 1997 [8]. While similar, the prevalence values from other studies were measuring prenatal use of only herbal medicines as opposed to this study which measured the use of traditional remedies as well as consultation of traditional practitioners. This prevalence rate of 52% is not necessarily representative of the whole of Zimbabwe because the selection of study sites was not random and representative of Zimbabwe. Rather, the sites were chosen only because they were the same sites at which the NASG clinical trial is being conducted. The indications for use reported by women in this study differed from those in reviewed literature. The primary goal for the participants in this study seemed to be the prevention of perineal lacerations, labour augmentation and achieving a safe delivery process while the motivation for use by women from Western countries was to treat conditions precipitated by pregnancy, to supplement their nutritional supply and to treat the same conditions usually treated by conventional medicines [6]. Other indications reported in literature were; increasing sexual pleasure [3], spiritual cleansing [5] and also for lactation [9]. Because data collection was based on what the interviewees reported, the study was vulnerable to recall bias and possibly deliberate incorrect answers in cases where the correct answer could be incriminating to the participant. Only two study participants reported using *Cannabis sativa (mbanje) -* based TM for labour induction. The investigator had anticipated this number to be higher considering that the conductors of the NASG clinical trial had implied that their subjects reported its use frequently. Because use of *Cannabis sativa* is an illicit practice according to the Medicines and Allied Substances Control Act Chapter 15:03 of Zimbabwe, participants could have been deterred from reporting that they used it regardless of the confidentially agreement.

The study population was almost homogeneous as far as demographic characteristics were concerned. All except three participants cited Christianity as their religion and all reported that they had received at least primary level education with the majority (86%) reporting that they had received education only up to the secondary level. The mere location of the study sites meant that the women available for recruitment all resided in high density residential areas, which might limit generalizability of the results. The most commonly used TM interventions reported in this study were not consistent with those reported by any of the literature reviewed prior to data collection. This emphasized the need for every region to perform its own study because results from different parts of the world were not necessarily analogous. Nulliparity and nulligravidity were found to be significantly associated with prenatal use of TM, possibly because without prior experience, nullipara women were prone to accepting advice concerning their pregnancy. Nulliparity and nulligravidity were also predisposing factors to experiencing adverse events, mostly perineal tears, just as Lindgren and co-workers highlighted [10]. The authors could not fully establish why residing in the Mbare neighbourhood was significantly associated with prenatal use of TM. However, it could be postulated to be due to the close proximity of Mbare to Harare’s largest population of informal vendors, including TM vendors (Mbare Musika).

## Conclusions

Based on the findings of this study, it was concluded that, the prevalence of prenatal use of traditional medicine among women seen at the selected Harare clinics was 52% at a standard error of 3.17%. Of these users, the majority (N = 96) used a single intervention while others used two or more. Pregnant women in Harare’s high density residential areas used TM practices to induce labour, avoid perineal tearing and improve the safety of their delivery process.

This study alone did not address the query which the NASG Clinical Trial team raised concerning a possible link between excessive postpartum bleeding and prenatal use of traditional medicine. However, because the most commonly used TM interventions were identified in this study, further extensive literature searches and in-vitro studies could follow. Characteristics of pregnant women which were significantly associated with prenatal use of TM were; being in the 20–25 age group (p = 0.021), nulliparity (p = 0.004), nulligravidity (p = 0.002), and residing in Mbare (p = 0.046). Statistically, the tribe of origin, religion and level of education and employment status were not significantly associated with prenatal use of TM. Non-use of TM, nulliparity and nulligravidity were found to be significantly associated with experiencing adverse events while use of TM was not. Because prenatal use of TM was fairly common practice while those participants who claimed non-use of TM reported experiencing more adverse events, further confirmatory studies with more refined designs such as case–control, cohort or animal studies would be warranted. Such studies would determine which medicines, if any, were protective against or causative of obstetric adverse events, hence establish the possible mechanism of action and provide the necessary clinical evidence for encouraging or discouraging the prenatal use of TM.

## Competing interests

There were no competing interests for any of the authors.

## Authors’ contributions

CCM made substantial contributions to the conception and design of the study and gave final approval for the version to be published. TGM was involved in the design of the study and revising the manuscript critically for important intellectual content. DDM collected data, analysed and interpreted it, funded the study and drafted the manuscript. All authors read and approved the final manuscript.

## Pre-publication history

The pre-publication history for this paper can be accessed here:

http://www.biomedcentral.com/1472-6882/12/164/prepub
